# Autodissemination stations suppress *Aedes notoscriptus* mosquitoes and reduce Buruli ulcer risk in urban Australia: a randomized controlled field trial

**DOI:** 10.1038/s41564-026-02439-8

**Published:** 2026-08-27

**Authors:** Véronique Paris, Andrew H. Buultjens, Nicholas Bell, Thomas L. Schmidt, Wendy M. Bamberg, Michael T. Birrell, Finn Romanes, Catarina A. Antao, Maria Globan, Caroline Lavender, Katherine Bond, Sally Dougall, Jake A. Lacey, Yichen Zhai, Peihong Guo, Norelle L. Sherry, Katherine B. Gibney, Jue Tao Lim, Paul D. R. Johnson, Timothy P. Stinear, Ary A. Hoffmann

**Affiliations:** 1 Pest and Environmental Adaptation Research Group, School of BioSciences, Bio21 Institute, University of Melbourne, Parkville, Victoria Australia; 2 Department of Microbiology and Immunology, Doherty Institute, University of Melbourne, Melbourne, Victoria Australia; 3 School of Life and Environmental Sciences, University of Sydney, Camperdown, New South Wales, Australia; 4 Western Public Health Unit, Western Health, St Albans, Victoria Australia; 5 Department of Health, Melbourne, Victoria Australia; 6 WHO Collaborating Centre for Mycobacterium ulcerans, Mycobacterium Reference Laboratory, Victorian Infectious Diseases Reference Laboratory, Doherty Institute, Melbourne, Victoria Australia; 7 Microbiological Diagnostic Unit Public Health Laboratory, Department of Microbiology and Immunology, Doherty Institute, University of Melbourne, Melbourne, Victoria Australia; 8 Lee Kong Chian School of Medicine, Nanyang Technological University, Singapore, Singapore; 9 Department of Infectious Diseases and Immunology, Austin Health, Heidelberg, Victoria Australia; 10 WHO Collaborating Centre for Antimicrobial Resistance, Doherty Institute, University of Melbourne, Melbourne, Victoria Australia; 11 Victorian Infectious Diseases Service, Royal Melbourne Hospital, Doherty Institute, Melbourne, Victoria Australia; 12 Department of Infectious Diseases, University of Melbourne, Doherty Institute, University of Melbourne, Melbourne, Victoria Australia; 13 Centre for Pathogen Genomics, University of Melbourne, Doherty Institute, University of Melbourne, Melbourne, Victoria Australia

**Keywords:** Bacterial infection, Ecological epidemiology, Pathogens

## Abstract

*Aedes notoscriptus* are mosquito vectors implicated in transmission of *Mycobacterium ulcerans*. This bacterium causes a destructive infection of skin and soft tissue called Buruli ulcer. Here we ran a randomized controlled trial in an urban Buruli ulcer endemic area in Melbourne, Australia to test whether autodissemination mosquito control stations, containing pyriproxyfen (larvicide) and *Beauveria bassiana* (entomopathogenic fungus), suppress *Ae. notoscriptus* populations. Six geographic areas each received 100 autodissemination stations for 8 weeks, and six control areas received no stations between 25 January 2024 and 21 March 2024. The primary outcome measure was mosquito population numbers. After the trial, there was a 70% average reduction in mosquito egg counts among the six intervention areas compared to control areas (*P* = 0.0076). In an ad hoc analysis, we then explored human Buruli ulcer notifications in treatment and control areas. After accounting for the 4.8-month mean incubation period, there was an 83% reduction in infection likelihood coinciding with peak intervention effect (intervention zones 1 case, control zones 6 cases, incidence ratio rate 0.167, 95% CI 0.0026–1.054, *P* = 0.047). The effect was not observed during the same time period in the year previous or following 2024, when no interventions were undertaken. A strong correlation (*R*^2^ = 0.85) was observed between decreased disease risk and mosquito suppression. These data show that autodissemination traps can effectively lower urban mosquito populations and reduce the threat of Buruli ulcer in humans.

## Abstract (web summary)

A randomized controlled trial shows that autodissemination stations reduce egg counts for *Aedes notoscriptus*, the *Mycobacterium ulcerans* vector, with exploratory analyses suggesting that the intervention could also reduce human Buruli ulcer cases caused by these bacteria, in intervention areas in Melbourne, Australia.

## Main

Buruli ulcer is a destructive infection of skin and soft tissue caused by *Mycobacterium ulcerans*. The infection is geographically restricted and acquired from the environment. In the temperate southeast of Australia in the state of Victoria, there has been a dramatic rise in cases of Buruli ulcer that is now reaching into inner suburbs of the capital city Melbourne (population 5,200,000) and the regional centre of Geelong (population 280,000). The incidence in Victoria was 5.8/100,000 population in 2024 and there are routinely more than 350 cases per year^1^. However, as Buruli ulcer is transmitted in very restricted areas, the local annual incidence and impact can be much higher; 770/100,000 in one coastal town^1^. In temperate Victoria, it has now been established that Buruli ulcer is a zoonosis of native possums that both develop skin ulcers and can excrete high concentrations of *M. ulcerans* in their faeces^2–5^. Humans are spillover hosts who are exposed to mosquitoes in endemic areas during the mosquito season in summer and autumn but who typically do not develop overt disease until winter and early spring, a paradox explained by the long incubation period^6,7^. The mean incubation period for Buruli ulcer is 4.8 months with an interquartile range (IQR) of 101–171 days^6^.

Recent research has shown that *M. ulcerans* transmission is associated with *Aedes notoscriptus*, an Australian mosquito species that thrives across a wide range of climates and habitats and is common in residential backyards^8^. This distribution is closely tied to its tendency to breed in small containers^9^. In urban settings, it displays a strong preference for feeding on humans but will also target other animals, including possums^8^. Notably, *Ae. notoscriptus* has greater dispersal capacity than other container-inhabiting *Aedes*^10–12^, potentially making localized control efforts challenging. The association with urban environments helps explain this mosquito’s role as an important vector for several zoonotic diseases including Ross River virus infection^13^, Barmah Forest virus^14^ infection, and most recently, Buruli ulcer^8^. This mosquito species is also a substantial vector of dog heartworm^15^.

New tools have recently emerged to suppress mosquito populations, including the In2Care™ Mosquito Station (https://www.in2care.org/), which targets both immature and adult mosquitoes in a dual-action approach. The development of immature mosquitoes within the station is disrupted by pyriproxyfen, an insect growth regulator, which is then also disseminated by adult mosquitoes to untreated breeding sites that include cryptic sites not easily treated directly. In addition, adult mosquitoes are infected with *Beauveria bassiana*, an entomopathogenic fungus that eventually kills them. The efficacy of In2Care stations has been validated for several mosquito species in a range of different settings, including *Ae. aegypti*, *Ae. albopictus* and *Culex quinquefasciatus*^16–19^. However, outcomes are influenced by how closely deployment aligns with recommended practices and local environmental contexts. For instance, deployment of a low density of stations failed to effectively reduce *Ae. aegypti* and *Cx. quinquefasciatus* populations in Florida^18^. A trial in Hawaii also failed to reduce *Ae. albopictus*, probably due to an abundance of alternative breeding sources, limiting mosquito visitation to the stations and reducing autodissemination effects^20^. Evidence that reductions in mosquito numbers achieved through autodissemination approaches translate into decreased disease transmission remains limited and is largely derived from non-randomized or observational studies^21–23^.

Systematic studies over 20 years have established that mosquitoes are the principal vector of *M. ulcerans* in Victoria^8^. However, there have not yet been any attempts to reduce human Buruli ulcer incidence by controlling local mosquitoes. To address this issue, we conducted a randomized controlled trial of the In2Care mosquito control station^16,24^ and assessed the effect on mosquito numbers (primary outcome) and human Buruli ulcer cases (secondary outcome). This study followed on from preliminary trials near Melbourne, Australia, which indicated a high efficacy of In2Care stations against *Ae. notoscriptus* when deployed at the recommended density^24^.

Here we extend our previous research and present a larger-scale, randomized controlled field trial of the In2Care system. We show that these autodissemination traps significantly reduced mosquito populations and subsequently lowered the risk of human Buruli ulcer.

## Results

### Study design and trial site selection

**Fig. 1:**
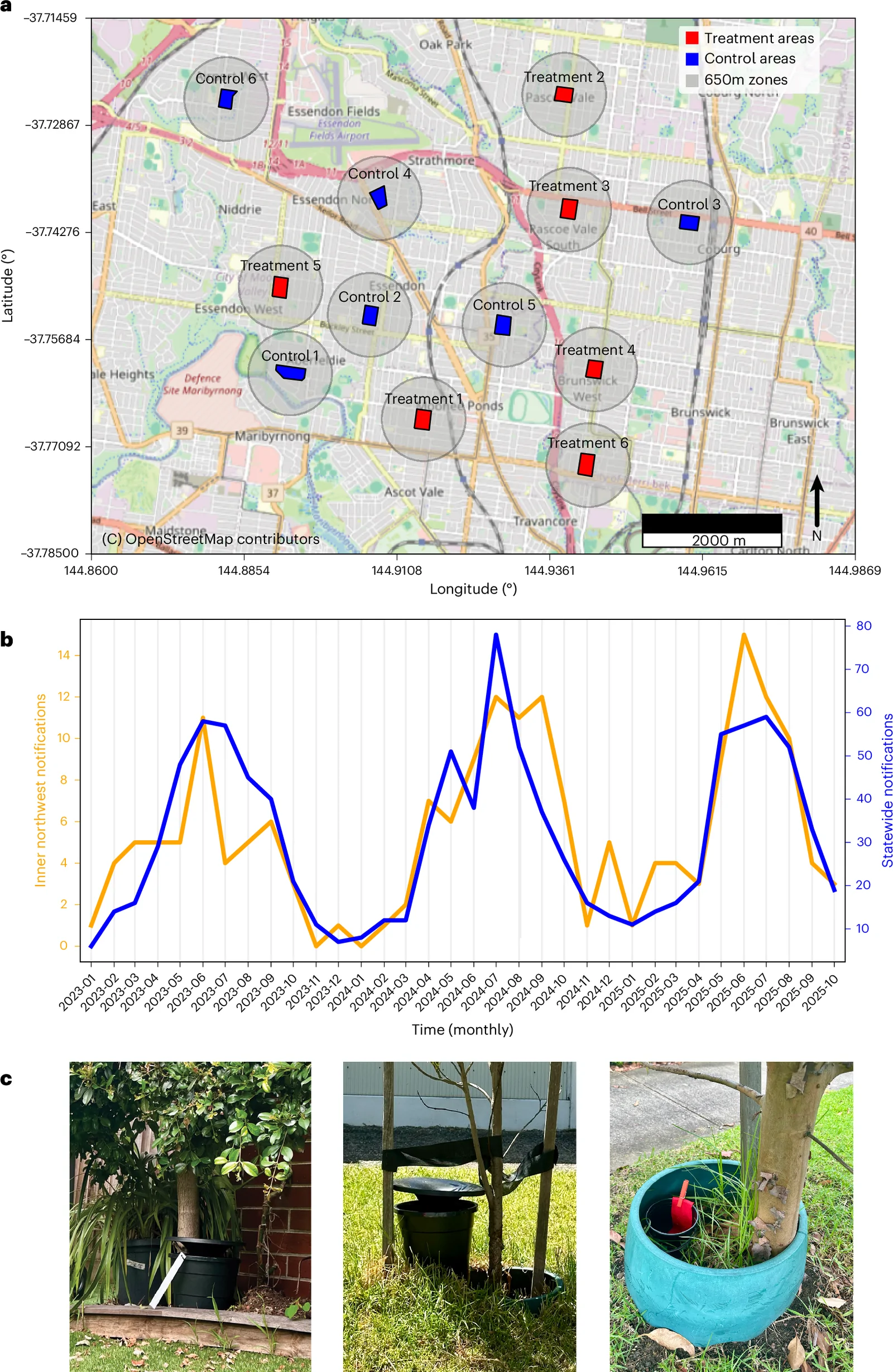
Randomized controlled trial design of the intervention study. **a**, Map of the inner northwest region showing the location of the randomly assigned treatment areas (red rectangles) and control areas (blue rectangles) with 650 m radius zones around each area based on *Ae. notoscriptus* range. **b**, Epidemic curve for human Buruli ulcer case notifications over 3 calendar years (Jan 2023–Nov 2025) in the 50 km^2^ study region (left *Y* axis) and the entire state of Victoria (right *Y* axis). Due to the long incubation period, transmission in summer results in notification peaks in the winter months as shown. **c**, Images of the study area, showing examples of deployment of the In2Care mosquito control stations (left and middle) and the ovitraps for mosquito egg counting (right).

In this interventional study, we selected a 50 km^2^ area across inner northwestern suburbs of Melbourne, Victoria, Australia with active Buruli ulcer transmission based on 2023 human case data^25^ (Fig. 1a,b and Supplementary Table 1). After matching 12 areas across the region (Supplementary Table 2), we used a random number generator to assign 6 treatment areas and 6 control areas. Each area comprised ~80 residential houses (Fig. 1a). Approximately 100 In2Care mosquito autodissemination stations were deployed in each treatment area for a period of 8 weeks from 25 January 2024 to 21 March 2024. The primary outcome aim of the intervention was to test whether the In2Care stations could significantly reduce *Ae. notoscriptus* populations in an urban setting (Fig. 1c).

### Mosquito egg number comparisons before and after the trial

**Fig. 2:**
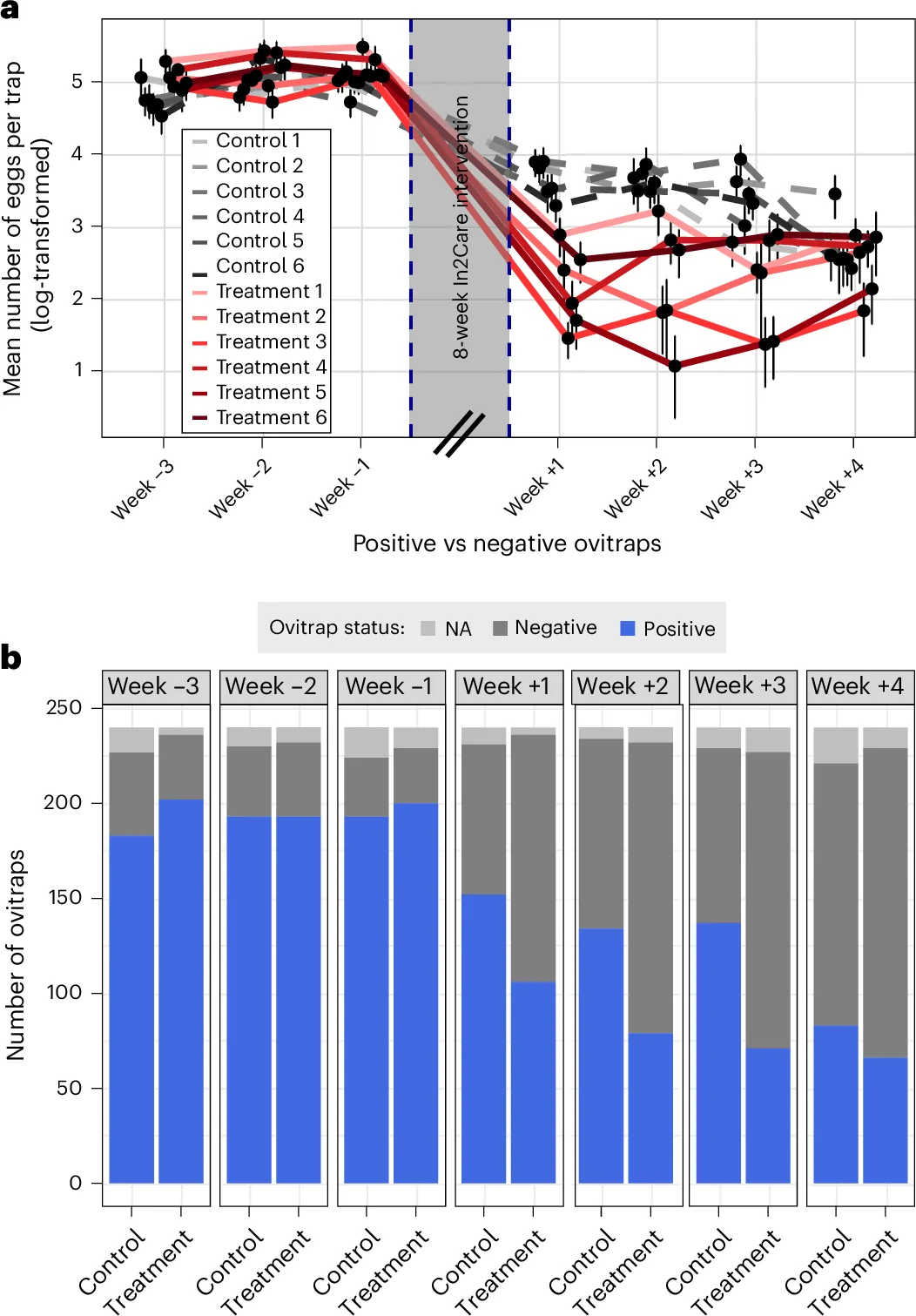
Comparison of mosquito egg counts between treatment and control areas. **a**, Weekly mean egg counts in the treatment sites (red solid lines) and in control areas (grey dashed lines). Egg counts were averaged from *n* = 40 ovitraps per site per week; black error bars show standard errors. Egg counts were not obtained during the intervention to avoid competition between ovitraps and In2Care stations. Statistical comparisons were performed using two-sided GLMMs with a negative binomial distribution, with sampling area, sampling week and their interaction as fixed effects, and trap-level heterogeneity as a random effect; model selection was based on lowest AIC. Egg counts were significantly influenced by sampling area and sampling week (*z* = 78.05, *P* < 0.001). **b**, Proportion of positive ovitraps (collected eggs, blue bars), negative ovitraps (no eggs, dark grey bars) and unrecoverable ovitraps (NA, light grey) in control and treatment areas before and after the intervention. *n* = 40 traps per area. Statistical comparisons were performed using two-sided GLMMs with sampling week and treatment area as fixed effects, and trap-level heterogeneity as a random effect. Sampling week and treatment area significantly influenced ovitrap positivity (*z* = 6.208, *P* < 0.001).

Before deployment of the stations (hereafter called ‘the intervention’), we assessed the overall distribution of mosquitoes across the treatment and control areas by monitoring egg counts from 480 ovitraps distributed across the study area in a 3-week period before the intervention and a 4-week period after the intervention concluded (Supplementary Table 3). Egg counts were highly variable across clusters and sampling weeks, with coefficients of variation (c.v. = s.d./mean) for raw counts >1 in all weeks and often >2 in post-intervention weeks when mean egg numbers were low. C.v. calculated on log-transformed counts were substantially lower, particularly in the pre-intervention period, indicating reduced relative dispersion on the transformed scale, although variability remained high in later post-intervention weeks. We first fitted a generalized linear mixed model (GLMM) that indicated the number of eggs per trap was influenced by the sampling area as well as the sampling week (*z* = 78.05, *P* < 0.001). Post hoc comparisons showed no significant differences in egg counts between the control and treatment areas in the 3 weeks of baseline egg counting undertaken before the intervention. The first sampling after the 8-week intervention was completed, showed significantly lower egg counts at all treatment areas compared with all control areas (Fig. 2a and Supplementary Table 3). This trend continued in the second week of egg counts after the intervention. Egg numbers at treatment areas started to merge with control area egg counts in weeks 3 and 4 after the cessation of the intervention (Supplementary Table 3, and Fig. 2a).

### Comparison of positive versus negative ovitraps

We tested whether the number of positive and negative ovitraps changed over the course of the intervention, considering both sampling week and treatment group. The sampling week and treatment area significantly influenced the number of positive ovitraps (*z* = 6.208, *P* < 0.001).

Before the In2Care intervention, ~80% of ovitraps across all sampling areas were positive, with no significant difference between treatment and control areas (treatment areas before intervention vs control areas before intervention: *z* = −1.664, *P* = 0.343). After the intervention, the proportion of positive ovitraps in the treatment areas significantly decreased to 33.4% (treatment areas before the intervention vs treatment areas after the intervention: *z* = 18.588, *P* < 0.001). In comparison, control areas showed a smaller reduction in positive ovitraps, with 52.7% positive after the intervention (control areas before the intervention vs control areas after the intervention: *z* = 11.041, *P* < 0.001). In addition, the difference between control and treatment areas after the intervention was highly significant (control areas after the intervention vs treatment areas after the intervention: *z* = 7.844, *P* < 0.001), indicating that the intervention effectively reduced the number of positive ovitraps in treatment zones relative to controls (Fig. 2b)

### Synthetic control egg number comparison

**Extended Data Fig. 1:**
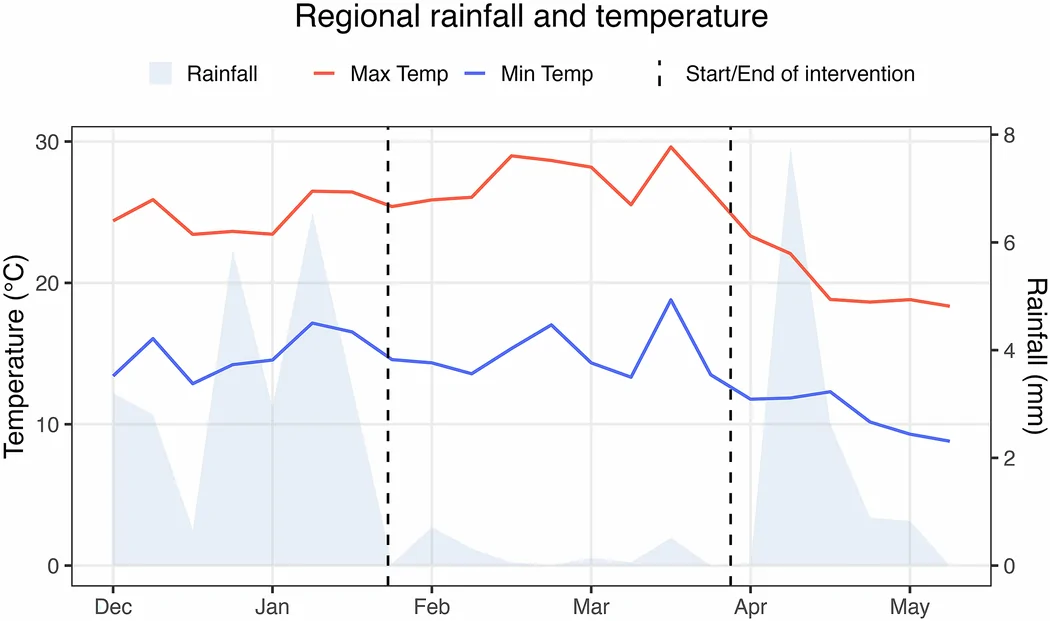
Weekly mean regional temperature and rainfall data. Minimum and maximum temperature are shown as red and blue lines respectively. Mean cumulative rainfall is represented by the area plot (light blue). Weather data were obtained from the Bureau of Meteorology (bom.gov.au) Melbourne Essendon Airport station. The vertical dashed lines indicate the start and end dates of the intervention.

The impact of the intervention was confirmed with an additional analysis that compared egg numbers from each treatment area to a synthetic control (average egg count from all control areas), showing that a significant decrease in mosquito numbers at all treatment areas persisted 2 weeks after the intervention concluded (Table 1). There was a 70.3% decrease in average egg numbers across all treatment areas, compared to all control areas in the 3 weeks after intervention. A period of very low rainfall during the intervention explains the overall reduction in egg numbers across all areas (see following section) (Fig. 2a and Extended Data Fig. 1).

**Table 1:** Impact of the intervention on mosquito egg numbers at each treatment site compared to synthetic control

Week	Area	*t* value	d.f.	*P* value
Before intervention				
Week −3	Treatment 1	2.3434	39	0.0243*
	Treatment 2	1.1735	38	0.2479
	Treatment 3	0.8218	37	0.4165
	Treatment 4	1.8279	38	0.07542
	Treatment 5	0.4776	39	0.6356
	Treatment 6	0.9571	39	0.3444
Week −2	Treatment 1	2.1405	38	0.03879*
	Treatment 2	−0.4938	39	0.6242
	Treatment 3	−1.8503	37	0.07227
	Treatment 4	1.84	38	0.07359
	Treatment 5	0.7379	36	0.4654
	Treatment 6	0.949	38	0.3486
Week −1	Treatment 1	3.0218	38	0.00448*
	Treatment 2	0.4092	36	0.6848
	Treatment 3	0.4825	37	0.6323
	Treatment 4	1.3088	37	0.1987
	Treatment 5	0.4296	37	0.67
	Treatment 6	0.3912	38	0.6979
After intervention				
Week +1	Treatment 1	−1.8946	38	0.06577*
	Treatment 2	−12.089	38	1.36 × 10^−14^^*^
	Treatment 3	−9.5556	37	1.56 × 10^−^^11*^
	Treatment 4	−4.6034	36	5.01 × 10^−^^05*^
	Treatment 5	−23.573	38	< 2.2 × 10^−^^16*^
	Treatment 6	−5.1407	39	8.03 × 10^−^^06*^
Week +2	Treatment 1	−6.3669	36	2.25 × 10^−^^07*^
	Treatment 2	−3.1452	38	0.00322*
	Treatment 3	−15.061	33	< 2.2 × 10^−^^16*^
	Treatment 4	−2.6077	35	0.01331*
	Treatment 5	−15.787	39	< 2.2 × 10^−^^16^^*^
	Treatment 6	−2.1549	39	0.0374*
Week +3	Treatment 1	0.4215	37	0.6758
	Treatment 2	−0.3828	37	0.7041
	Treatment 3	−3.2892	39	0.00214*
	Treatment 4	−0.1907	34	0.8499
	Treatment 5	−2.2653	39	0.02912*
	Treatment 6	0.2026	37	0.8406

*Egg count differences reaching statistical significance.

### Environmental factors impacting egg counts

During the intervention, the average maximum temperature was 25.1 °C, the average minimum temperature was 15.4 °C, and the average rainfall was 13.9 mm. Rainfall was a significant positive predictor of mosquito egg counts (estimate = 0.032, s.e. = 0.0056, *t* = 5.74, d.f. = 69, *P* < 0.001), as was minimum temperature (estimate = 0.156, s.e. = 0.051, *t* = 3.03, d.f. = 69, *P* = 0.003). In contrast, maximum temperature had a significant negative effect on egg counts (estimate = −0.132, s.e. = 0.018, *t* = −7.47, d.f. = 69, *P* < 0.001). Green cover, number of houses and area (km^2^) were not significant predictors in the model (all *P* > 0.1). Including zone as a random effect improved model fit, accounting for modest between-area variability.

### Breeding site hotspot analysis

**Extended Data Fig. 2:**
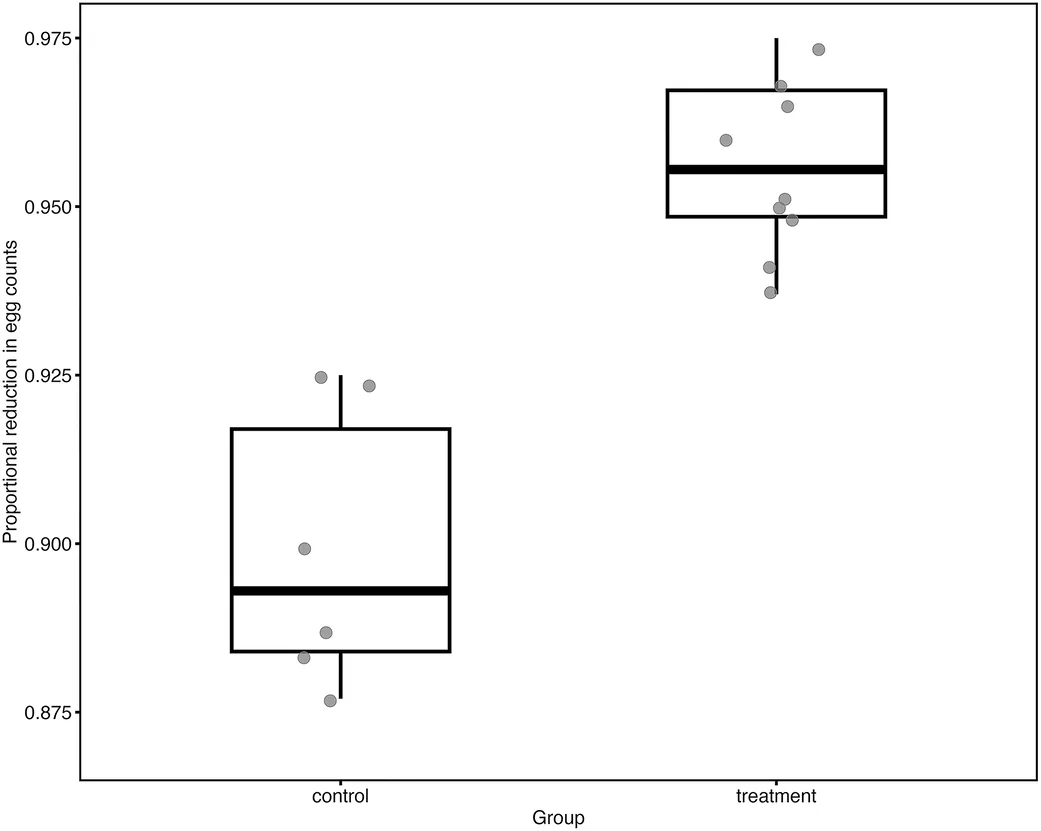
Proportional reduction in egg counts. Comparison between control and treatment (intervention) clusters, based on the 10 traps with the highest pre-intervention counts in each cluster. The y-axis represents the proportional reduction in egg counts (pre-intervention minus post-intervention / pre-intervention), while the x-axis distinguishes between the control and treatment groups. Points represent individual site reductions, and boxplot elements show the median, interquartile range, and variability.

The 10 traps with the highest initial egg counts exhibited substantial declines in egg counts in both control and treatment areas (Extended Data Fig. 2). Mean egg counts in hotspot traps declined from 672 to 21 eggs per trap in treatment areas (96.9% reduction) and from 491 to 34 eggs per trap in control areas (93.0% reduction) reflecting strong post-intervention declines in oviposition overall. Despite these large reductions in both groups, the magnitude of decline was significantly greater in treatment areas (Welch’s *t*-test, *t* = −3.25, d.f. = 12.17, *P* = 0.007). The mean absolute reduction in egg counts was 651.3 in the treatment group compared to 457.1 in the control group (95% CI for difference: −324.4 to −64.1), indicating that In2Care stations more effectively reduced oviposition in mosquito breeding hotspots.

### Assessing intervention effect on human Buruli ulcer

Given the success of the In2Care stations for reducing mosquito numbers, we were curious to see whether there had been any impact of this intervention on human Buruli ulcer cases in treatment versus control areas. A standard approach to address this question in randomized controlled trials of vector reduction interventions is to compare disease incidence in the treatment areas with disease incidence in the control areas using an a priori defined set of conditions and analysis methods^26^. However, this trial was designed and powered to test the efficacy of the In2Care stations for mosquito control and only post hoc analyses of state Health Department Buruli ulcer case notification data were available to explore any potential intervention impact on human cases. The epidemiology in the study area for this 3-year period and region was consistent with other Victorian endemic areas and the established 138-day mean incubation period (IQR 71 days), with peak case notifications reported in the winter and spring months (Fig. 1b)^6,7^. The long incubation period and small study area with relatively low number of cases (66 cases in overall region reported for 2024^25^) posed challenges for assessing intervention impact on disease transmission.

Further challenges included uncertain exposure locations and dates. However, by using *M. ulcerans* genome sequence data from bacterial isolates cultured from Buruli ulcer patients, we were able to show that cases were acquired in the study area. By reference to the *M. ulcerans* core genome phylogeny, we confirmed that these cases were infected with the *M. ulcerans* genotype attributable with high confidence to this specific region of Melbourne^27^ (Supplementary Fig. 1 and Tables 5 and 8). Further, from previous genomic epidemiological studies of familial Buruli ulcer case clusters, we know that infections are most probably acquired around the home^28^.

**Fig. 3:**
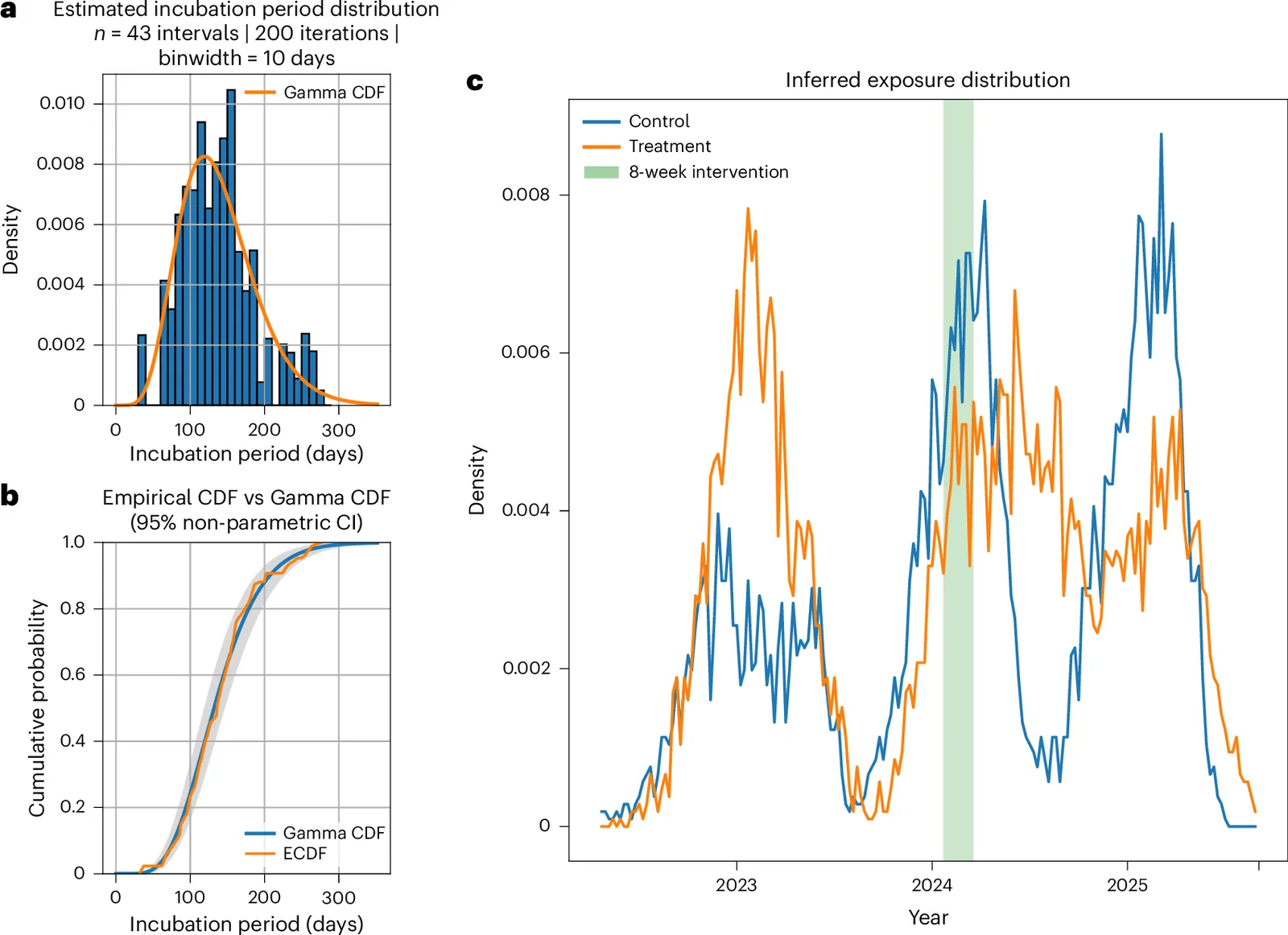
Assessing the impact of intervention on Buruli ulcer incidence. **a**, Gamma distribution fitted to observed incubation period from point exposure Buruli ulcer cases^6,42^. **b**, Cumulative probability based on empirical and gamma distribution functions of the observed incubation periods. CDF, cumulative distribution function. **c**, Patterns of likely exposure dates based on 200 repeated draws from the fitted gamma distribution, with peaks annotated (Extended Data Fig. 2). Green shading indicates 8-week In2Care trial period.

Our first approach to account for the uncertain exposure dates was to work backwards from symptom-onset dates to reconstruct when infections most probably occurred (Fig. 3). To do this, we fitted the established point exposure data to a gamma distribution (Fig. 3b,c)^6,7^. Cases were classified into either a treatment or control zone if the patient residential address (mesh block centroid) was located within 650 m of the corresponding treatment or control area centroid (Fig. 1a).

For each case, we repeatedly drew a possible incubation time from this probability distribution and subtracted that time from the known symptom-onset date. The collection of these iterated exposure dates for 2023, 2024 and 2025 formed a probabilistic estimate of when a person was infected across each year (Fig. 3c). We then used these data to infer Buruli ulcer incidence during the 8-week intervention period but observed no significant difference in relative risk (RR) between treatment and control zones (RR = 1.023, 95% CI 0.874–1.231) (Fig. 3c).

**Fig. 4:**
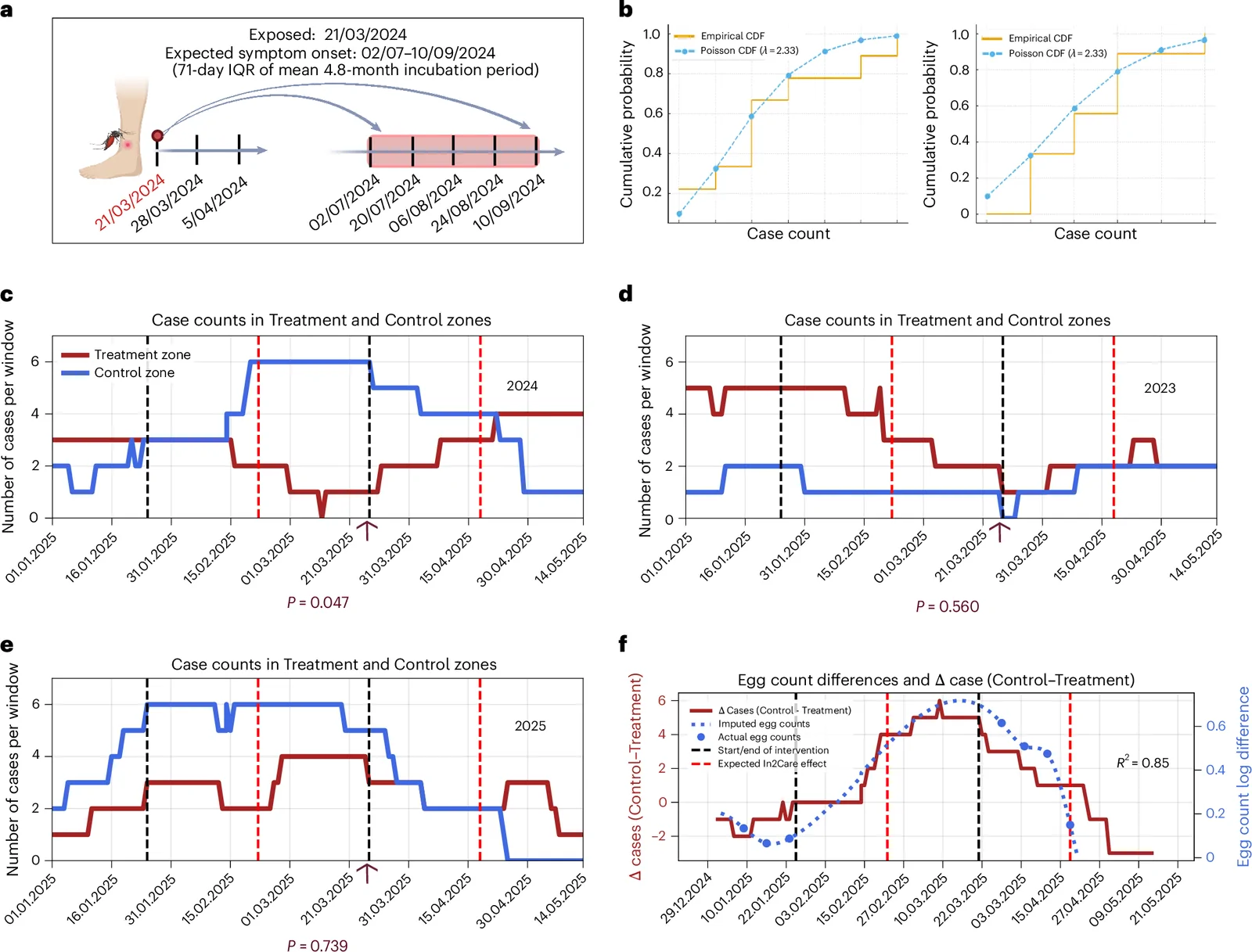
Comparison of Buruli ulcer case notifications and mosquito population size between treatment and control zones. **a**, Overview of 71-day IQR and sliding-window analysis to assess the likely *M. ulcerans* exposure period. Example shows a Buruli ulcer case exposed 21/03/2024, after which there is a 50% likelihood that symptom onset would have occurred between 02/07/2024 and 10/09/2024. Created in BioRender: Stinear, T. https://BioRender.com/vzdu7ld (2026). **b**, Kolmogorov–Smirnov goodness-of-fit tests were used to assess whether case counts followed a Poisson distribution. No significant deviation from a Poisson distribution was detected for either treatment zones (left; *P* = 0.245) or control zones (right; *P* = 0.528). **c**, Sliding-window profile of Buruli ulcer case notification reported within the symptom-onset period 101–171 days following each of 136 exposure windows that occurred within treatment or control zones. Annotated are the date of predicted peak impact (arrow) of In2Care stations on mosquito reduction (21/03/2024) and *P* value from a two-sided Poisson likelihood ratio test comparing case counts between treatment and control zones for the highlighted exposure window (no adjustment for multiple comparisons was applied). Black and red vertical dashed lines denote the 8-week In2Care intervention period and the expected period of In2Care effect, respectively. **d**,**e**, Same sliding-window profiles but for Buruli ulcer case notifications in 2023 and 2025, respectively. **f**, log differences in mosquito egg counts between control and treatment zones shown with difference in case numbers between control and treatment zones across the sliding exposure windows. Observed egg count differences are indicated by dots, while imputed values are depicted with a dotted line. Black and red vertical dashed lines denote the 8-week In2Care intervention period and the expected period of In2Care effect, respectively.

**Extended Data Fig. 3:**
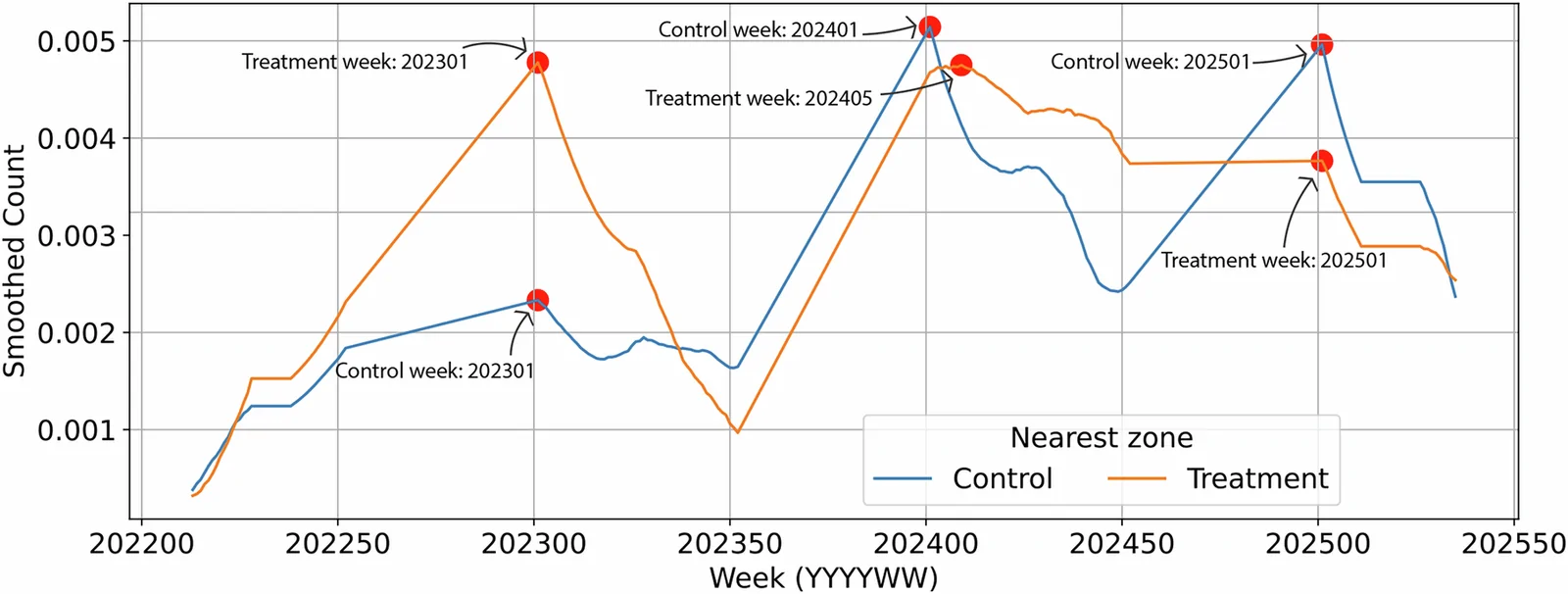
Seasonal timing of Buruli ulcer exposure peaks in treatment and control zones inferred from Monte Carlo incubation modelling. Weekly Buruli ulcer case densities in treatment (orange) and control (blue) zones inferred from Monte Carlo–reconstructed exposure dates (Fig. 3). Weekly counts were smoothed using a 50-week centred moving average. For each year (2023–2025), the dominant seasonal peak was identified as the week of maximum smoothed case density and is marked with coloured circles and annotated by year and week (YYYYWW).

However, we observed in our exposure inferences (Fig. 3c) that the peak probability of Buruli ulcer infection in the treatment zones was delayed by +28 days compared to the inferred peak probability in control zones during the intervention, an offset not observed in the preceding and subsequent year (2023 and 2025, when it was 0 days) when no mosquito control interventions were undertaken (Fig. 3c and Extended Data Fig. 3). To test the hypothesis that the intervention induced this delay, we compared the number of cases between control and treatment zones that were probably acquired during the predicted peak impact of the In2Care stations at week 8 of deployment (21 March 2024), a timepoint based on our previous research^24^. Here we used the 138-day mean and 71-day IQR of the incubation period, thus counting all cases with symptom onset reported between 2 July–10 September 2024 (Fig. 4a). Conceptually, a person infected on this day will have a 50% chance of experiencing symptom onset between 101 and 171 days later (because the IQR accounts for 50% of the data within the incubation period probability distribution).

We then compared disease incidence between treatment and control zones for this timepoint. As we used a Poisson likelihood ratio test for this comparison, case counts were first confirmed as Poisson-distributed, with the number of zones treated as the exposure denominator (Fig. 4b). The null hypothesis assumed equal disease incidence across treatment and control zones, whereas the alternative hypothesis specified a difference in disease incidence between arms. A Poisson likelihood ratio test was appropriate as these data comprise rare, independent events and the objective was to compare incidence between treatment and control zones. We observed an incidence ratio rate (IRR) of 0.167 (95% CI 0.026–1.054, *P* = 0.047), meaning there was an 83% reduction in incidence in treatment zones to control zones for that specific IQR window (Table 2). Comparisons between zones for the same date in 2023 and 2025 showed no significant difference in IRR (Table 2). Similarly, comparisons using dates 70 days on either side of 21 March (the minimum temporal separation that ensured no overlap of cases counted within the symptom-onset periods) showed no significant difference in IRR (Table 2). These observations are consistent with the intervention reducing risk of Buruli ulcer transmission during the predicted peak effect of the In2Care intervention.

**Table 2:** Summary of Poisson likelihood ratio test statistics

Year	Date	No. cases Treatment	No. cases Control	Incidence ratio rate	95% CI^b^	*P* value^c^
2023	11 Jan	4	1	4.0	0.601–26.606	0.165
	21 Mar^a^	2	1	2.0	0.262–15.262	0.560
	30 May	2	2	1.0	0.177–5.665	1.0
2024	11 Jan	3	1	3.0	0.430–20.936	0.306
	21 Mar^a^	1	6	0.167	0.026–1.054	0.047
	30 May	3	1	3.0	0.430– 20.936	0.306
2025	11 Jan	1	3	0.333	0.048–2.326	0.306
	21 Mar^a^	4	5	0.800	0.233, 2.750	0.739
	30 May	1	0	n/a^d^	n/a^d^	0.239

^a^Expected peak of intervention effect.

^b^Confidence intervals are score-based for the rate ratio.

^c^*P* values are from two-sided Poisson likelihood ratio tests comparing case counts between treatment and control zones (no adjustment for multiple comparisons).

^d^Comparisons with zero cannot be calculated.

### Sliding-window analysis, correlation with mosquito reduction

We next explored how Buruli ulcer case counts changed over time and correlated with changing mosquito numbers in control and treatment zones by using a sliding-window approach. This method counted Buruli ulcer cases that occurred in daily increments across the IQR of the incubation period (Fig. 4a) and was intended to capture the potential dynamic impact of the intervention over time given the presence of time-dependent co-variables. These variables included ‘onset’ and ‘offset’ of mosquito In2Care station effectiveness and changes in overall mosquito numbers due to meteorological conditions, while accounting for the long incubation period. The analysis considered exposure days from 1 January 2024 to 14 May 2024 (total of 136 exposure days), corresponding with 136 IQR symptom-onset windows (Fig. 4c and Supplementary Table 6).

Among the 66 Buruli ulcer cases that occurred across the whole endemic region in 2024, 60 had symptom-onset dates that fell within the 136 IQR windows. Of these 60 cases, 9 occurred in control zones across the IQR exposure windows. The profile of counts-per-exposure-window for control zones peaked during the previously predicted peak-exposure period (Fig. 4c). In contrast, there were 7 Buruli ulcer cases in treatment zones across the exposure days. At face value, 9 versus 7 counts are not different, however, the profile of case-counts-per-window was inverse to the control zones during the expected peak In2Care station effect period. There was one exposure day within the intervention period (8 March 2024) representing 71 consecutive symptom-onset days in which zero cases were reported in treatment zones (Fig. 4c and Supplementary Table 7). This contrasts with the pattern in the control zones for this day when 6 cases were observed in the symptom-onset period (Fig. 4c and Supplementary Table 7).

If the difference in the profile of the sliding exposure windows between treatment and control zones was caused by the intervention, then these patterns should be unique to the intervention period in 2024. To test this, we assessed the same 8-week interval in 2023 and 2025, when no interventions were run. An inverse sliding-window profile between control and treatment zones was only observed in 2024 and not for the same date range in either 2023 or 2025 (Fig. 4d,e).

**Extended Data Fig. 4:**
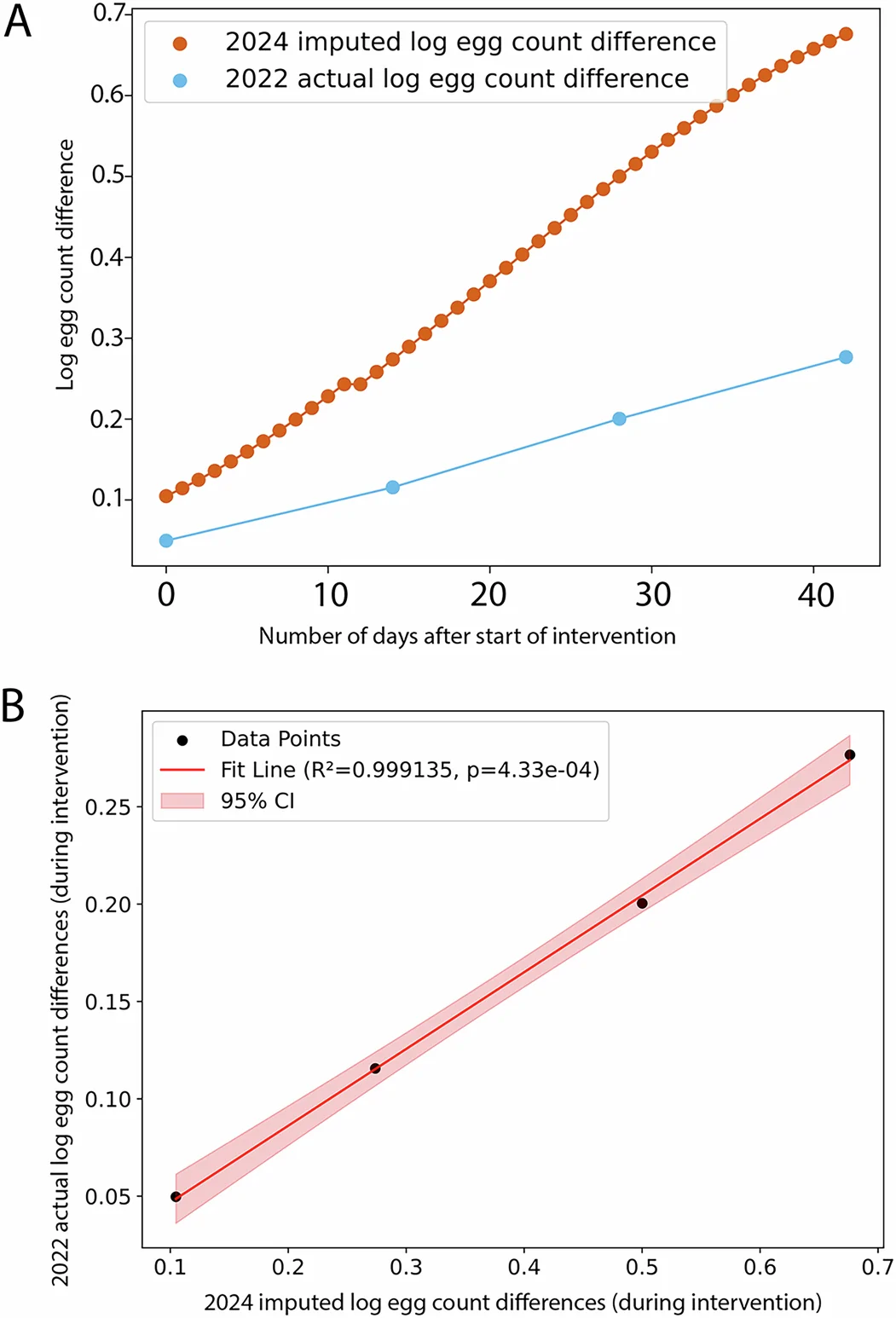
Validation of the imputation approach for missing egg count. (**A**) Time series plot showing the daily log egg count differences during the intervention. The x-axis represents the number of days since the start of the intervention. The red line (with daily data points) represents the 2024 imputed log egg count differences, while the blue line (with only four data points) represents the 2022 actual count data. (**B**) Linear model comparing the 2024 imputed and 2022 actual log egg count differences, using only the four days for which actual data were available. The model is annotated with the coefficient of determination (R^2^) and the p-value from a two-sided Wald test (t-distribution, n-2 degrees of freedom) testing whether the regression slope differs from zero.

Using the mosquito egg count data (Supplementary Table 2), we compared the changes in mosquito population size with the difference in Buruli ulcer cases between control and treatment zones across the exposure windows. We used imputation to estimate mosquito population changes during the intervention period when there was no egg monitoring. The imputation model was validated using observed data from a previous In2Care intervention conducted in a comparable local urban setting^24^ (*R*^2^ = 0.999, Extended Data Fig. 4). We then plotted the difference in cases between treatment and control zones across all exposure windows and compared that pattern with changes in mosquito egg counts before, during and after the intervention (Fig. 4f and Supplementary Table 6). The difference in cases tracked the reduction in mosquito egg counts across all exposure windows, consistent with a significant reduction in Buruli ulcer transmission risk attributable to the intervention (Fig. 4f). This association followed a positive linear trend (*R*^2^ = 0.85).

## Discussion

Accumulating evidence from southeastern Australia over the past two decades has shown *M. ulcerans* in association with mosquitoes and implicated them in the transmission of *M. ulcerans* to humans from a possum reservoir^4,8^. The current study reports an intervention that reduces the risk of human Buruli ulcer cases by specifically targeting local mosquito populations. This is an important finding because until now, public health authorities in Victoria have been unsuccessful in countering the rapid increase of Buruli ulcer incidence and its expansion into new endemic areas^29^.

Our primary motivation for this study was to test the effectiveness of the In2Care autodissemination control station for suppressing *Ae. notoscriptus* populations in urban environments, with a view to using this approach more broadly to support Buruli ulcer control activities. We chose a study area with active *M. ulcerans* transmission to subsequently obtain Buruli ulcer case notification data and assess the intervention impact on Buruli ulcer case numbers if we observed successful mosquito suppression using the In2Care stations. We focused on suppression of *Ae. notoscriptus* because of its specific association with *M. ulcerans* and Buruli ulcer in this region^8^. The 70% reduction in egg counts in intervention areas compared to control areas we observed after the 8-week trial confirms the effectiveness of autodissemination systems for mosquito population reduction in urban settings.

By targeting both adult mosquitoes and larvae, systems such as In2Care can complement traditional mosquito control methods, particularly in areas where cryptic mosquito breeding sites are common and when the target species exhibits skip oviposition behaviours as in *Ae. notoscriptus*^30^. Our findings also align well with previous studies demonstrating the effectiveness of pyriproxyfen-based autodissemination in reducing larval recruitment and overall population densities^24,31^. Assessment of the potential for mosquitoes to disperse pyriproxyfen into other parts of the ecosystem, where it could affect non-target insect species is warranted before widespread implementation^32^.

Although we have previously shown that native possums are a frequent bloodmeal source for *Ae. notoscriptus*, we have yet to establish whether *M. ulcerans* has a life cycle within mosquitoes or possums^8^. In this study we identified a clear linear relationship between reduction in mosquito egg numbers and reduced risk of Buruli ulcer in humans. This pattern supports a mechanical mode of transmission (Fig. 4) and suggests that any efforts to reduce mosquitoes and mosquito exposure are likely to be cumulatively beneficial, not requiring any specific threshold to be met to have a direct public health impact.

Limitations of this study include the short, 8-week intervention period and the post hoc rather than a priori analysis of the Buruli ulcer case notification data. To validate our findings, we assessed the 2023 and 2025 case data using the same statistical methods for the same 8-week period. A longer running trial, with a larger number of zones would be needed to adequately power future randomized controlled trials that have Buruli ulcer incidence reduction as a primary endpoint. Notwithstanding these factors, we show here that a mosquito intervention trial with a contemporaneous control arm significantly lowered *Ae. notoscriptus* populations and reduced the risk of Buruli ulcer in humans.

## Methods

### Ethics statement

Ethics approval for the use in this study of de-identified human Buruli ulcer case location, aggregated at the mesh block level, was obtained from the Victorian Government Department of Health Human Ethics Committee under HREC/54166/DHHS-2019-179235 and from the University of Melbourne Human Research Ethics Committee under 2024-31315-60974-3. Mesh blocks are the smallest geographic areas defined by the Australian Bureau of Statistics. They usually contain 30–60 dwellings. For household recruitment, the University of Melbourne Human Research Ethics Office confirmed (correspondence 5 Dec 2023) that ethics committee review was not required, as no personal data were collected and participation was voluntary.

### Permits

This work was performed under APVMA Permit number PER92912. Trials were conducted to generate data relating to efficacy, residues, crop or animal safety, or other scientific information outside the confines of a research facility where the site of the trial annually does not exceed the following: 1,000 In2Care stations over 40 hectares treated in total. The active ingredients in In2Mix sachets were imported under biosecurity permit 0006570560 from the Australian Government Department of Agriculture, Fisheries and Forestry.

### Study area

The 50 km^2^ study region included the Melbourne suburbs with the postcodes 3032, 3039, 3040, 3041, 3042, 3044, 3055 and 3058. Within this general region, 12 areas were chosen to be as comparable as possible with each other (for example, number of houses, sizes of residential blocks; Supplementary Table 2).

### In2Care stations

The In2Care mosquito station, made of polyethylene, consists of a lid, central tube, detachable interface and a reservoir holding 3.5–4.5 l of water with yeast tablets to attract gravid mosquitoes. A floating platform, equipped with a statically charged gauze strip coated with a powder containing pyriproxyfen (74%) and *B. bassiana* (10%), adjusts with the water level, providing a resting place for adult mosquitoes. When adult mosquitoes land on the gauze, the bioactives from the powder are transferred to the mosquitoes. After depositing eggs, adult mosquitoes leave the station and disseminate pyriproxyfen to other breeding sources. Pyriproxyfen inhibits the metamorphosis of mosquito pupae into adults and thereby prevents adult emergence, while *B. bassiana* causes mortality within 8–10 days. According to manufacturer recommendations, we placed one station per 400 m^2^ and serviced each one every 4–6 weeks by replacing the gauze strip and refreshing the reservoir with water, bioactive powder and yeast tablets (https://www.envu.com/Customers/Mosquito-Management/In2Care-Mosquito-Station).

### Mosquito monitoring

Population density of *Ae. notoscriptus* was evaluated using egg counts collected through ovitraps at control and treatment areas from 9 to 23 January 2024 to obtain an initial baseline and then again from 28 March until 18 April 2024 immediately following the removal of the traps. A total of 480 ovitraps (40 per site) were used, each consisting of a 500 ml bucket half-filled with water and alfalfa pellets to attract gravid mosquitoes (Fig. 1c). A 15-cm long and 7-cm-wide red felt strip provided an oviposition site. Eggs were counted weekly using a light microscope by the same researcher for consistency (Supplementary Table 3). To prevent breeding, all larvae and pupae were discarded at each collection. Egg counting was not performed during the intervention itself to avoid competition with the In2Care traps.

### In2Care station deployment and servicing

Within the treatment areas, In2Care stations were placed in participating residential properties, as well as on roadside nature strips and other public land, with a density of approximately 1 station per 400 m^2^ following manufacturer recommendation. This resulted in a total deployment of 593 stations (~100 per treatment area) over an 8-week period from 25 January 2024 until 21 March 2024. The stations were strategically placed in shaded or semi-shaded areas that were expected to attract *Ae. notoscriptus* (Fig. 1c). Some stations had to be placed in less favourable locations to achieve an even spatial distribution of the stations. During deployment, we filled the reservoir with ~4.7 l of tap water, attached the biocide-treated netting to the floater, and placed it on the water. We then shook the prepackaged In2Mix sachet provided with the stations before opening it. The remaining biocide powder and odour tablets were added to the reservoir before sealing the lid.

### Meteorological information

Weather data were obtained from the Bureau of Meteorology (bom.gov.au) Essendon Airport Observation Station. Regular monitoring of the In2Care stations was conducted as required following weather events (storms, strong rainfalls, dry conditions) to check for signs of disturbance, ensuring that the water level was sufficient (that is, filled more than halfway) and the netting remained dry and intact. After the stations were in place for 4 weeks, the biocides and odour tablets in the stations were refreshed (from 20 to 23 February) using the In2Mix provided with the stations as described above.

### Community engagement and recruitment of residential backyards

The intervention was supported by a public outreach programme designed to inform residents about the project, promote source reduction practices and recruit residents to host one to five In2Care stations in their backyards (depending on size of the residential block). Communication began in early January 2024, a week before setting up ovitraps. Initial messaging introduced the purpose of the ovitraps, explained how they would be used, and outlined the timeframe for their deployment in the area.

Residents in the 6 treatment areas received information about the In2Care intervention starting from the first week of baseline trapping. Information covered the need for mosquito control, timing of the intervention, how the In2Care stations work, and how residents could participate by hosting stations. Weekly updates continued until deployment. Participating households received a handout with hosting dates and contact details. Once the intervention began, all 12 study sites received guidance on mosquito bite prevention, including breeding site reduction, protective clothing and insect repellent (Supplementary Figs. 1–3). Households across the 6 treatment zones were recruited through this outreach programme and systematic door-knocking by members of the research team. Residents were approached at their homes and provided with a verbal explanation of the study purpose, the nature of the intervention, and what participation would involve. Households that agreed to participate provided verbal consent and were enrolled in the trial. No formal recruitment target was set before commencement; all households that agreed to participate during the door-knocking campaign were enrolled. A total of 251 households were recruited across the 6 treatment zones, all of which completed the trial, with no withdrawals or dropouts. No compensation, payment or material incentives were provided to participants. In2Care stations were set up and retrieved by members of the research team.

### Statistical analyses of mosquito egg count data

#### Egg number comparison before and after the intervention

Statistical analyses were performed in RStudio (v.1.14)^33^. To test whether egg counts were changing across areas and time, we used generalized linear mixed models (GLMMs) with a negative binomial distribution to address overdispersion and zero inflation using the ‘glmmTMB’ package^34^. Sampling area and sampling week were used as fixed effects along with an interaction term between the two. Trap-level heterogeneity was also accounted for by using a random-effects term. We selected the best-fitting model by comparing the Akaike information criterion (AIC) of models including interactions to all nested models and the null model; the model returning the lowest AIC was selected. We compared variables using post hoc comparisons produced using the ‘emmeans’ package in R^35^. Relative variability in egg counts was summarized using the coefficient of variation (c.v. = s.d./mean), calculated for both raw egg counts and log-transformed counts to assess the effect dispersion of the data.

#### Comparison of positive vs negative ovitraps

To test whether the sampling week and sampling area significantly affected the number of negative ovitraps (that is, ovitraps with zero eggs), we fitted GLMMs for each variable, using sampling week and sampling area as the fixed effects, while trap-level heterogeneity was accounted for by using a random-effects term.

#### Synthetic control egg number comparison

We also ran an analysis using a synthetic control produced from the weekly average across all six control areas and tested whether each treatment area differed from the synthetic control for each sampling week using one-sample *t*-tests. We calculated the percentage reduction in egg numbers by comparing the average egg count across all treatment areas to the average across all control areas in the 3 weeks after trapping.

#### Effect of ecological variables on egg counts

We tested whether the number of mosquito eggs collected was influenced by green cover, the number of houses, zone area (km^2^), rainfall and temperature. Green cover was calculated using the ‘greenR’ package^36^ to derive the green view index (GVI) from Google satellite images, while the number of houses was counted using Google satellite imagery. Weather data, including rainfall and temperature, were obtained from the BOM station (bom.gov.au) at Essendon Airport. To account for repeated sampling across spatial areas (zones), we fitted a linear mixed-effects model (LMM) with a random effect for each area. Egg counts were averaged per area per week and log transformed to reduce overdispersion. The final model included green cover, number of houses, area (km^2^), rainfall, minimum temperature and maximum temperature as fixed effects, with area (treatment or control) included as a random effect.

#### Breeding site hotspot analysis

To evaluate the effects of In2Care stations on mosquito breeding hotspots within the treatment areas, we analysed the reduction in egg counts in the ten traps with the highest egg counts identified before the intervention in both treatment and control areas. The average egg counts for these traps were calculated for the pre- and post-intervention periods, and the reduction in egg counts was determined by subtracting the post-intervention mean from the pre-intervention mean for each trap. Reductions in egg counts between treatment and control areas were compared using Welch’s *t*-test.

#### Genomic analyses to confirm case source attribution

Given the long incubation period of Buruli ulcer and the challenge this poses for accurate epidemiological source attribution, we assessed existing pathogen genome sequence data to support source assignments for the 2024 inner northwest cases. Previous whole-genome sequencing studies have identified single nucleotide polymorphisms (SNPs) unique to *M. ulcerans* from the inner northwest, enabling distinction between locally acquired infections and those originating elsewhere^27^. In this study, Illumina sequence data obtained from 478 clinical isolates were mapped to the *M. ulcerans* reference chromosome JKD8049, and core genome SNPs were called using Snippy v.4.4.5 (minfrac 0.9 and mincov 10) (https://github.com/tseemann/snippy). A maximum-likelihood phylogenetic tree was inferred using FastTree v.2.1.10 (GTR model)^37^. The Newick tree file is available in GitHub at https://github.com/abuultjens/buruli-ulcer-mosquito-control-intervention/blob/main/478_inner_NW.FastTree-ML_ROOTED.nwk.

#### Buruli ulcer case notification data

Buruli ulcer has been a notifiable disease in Victoria since 2004. Diagnoses were made by polymerase chain reaction (PCR) from ulcer swabs or skin biopsies, with culture isolation also attempted for subsequent genome sequencing. Clinical laboratories notify the Victorian Department of Health (DH) using automated electronic systems. Notified cases and their treating clinicians are contacted by public health officers to determine symptom onset and likely place of exposure in the preceding 12 months. Human Buruli ulcer case notifications for 2024, along with cases’ residential addresses (their presumed exposure site), aggregated at mesh block level (the finest census data resolution available and designed to capture 30–60 dwellings), were provided by the DH. Cases were included in the analysis if patient residential address was located within the inner northwestern endemic region of Metropolitan Melbourne, providing that infection outside the local endemic area had been excluded by pathogen genomic epidemiology, that is, if an *M. ulcerans* genome sequence obtained from a patient isolate had a genotype not associated with the inner northwest area (Supplementary Fig. 1 and Tables 4 and 5)^38^. Disease incidence was calculated on the basis of annual case numbers and 2021 census population data (latest data at time of writing), both available at postcode level. The median difference between diagnosis date and symptom-onset date (46 days, range 4–214 days) was used to infer missing onset dates in the dataset (Supplementary Table 4). The mean incubation period for Buruli ulcer is 138 days with an IQR spanning 101–171 days^6^.

#### Spatial classification of cases into treatment and control zones

The Haversine distance was calculated between the centroid of each mesh block containing Buruli ulcer cases and the centroid of the nearest treatment or control area^39^. A radius of 650 m was used to classify a case as within either a treatment or a control zone, as this distance offered minimal overlap between nearby zones and aligns with the expected *Ae. notoscriptus* dispersal rates (Fig. 1a)^12^. Cases were classified as belonging to either a treatment or a control zone if their address (mesh block centroid) was located within the zone radius of the corresponding area centroid, and cases within overlapping zones were allocated on the basis of proximity to their nearest site.

#### Reconstruction of exposure dates using empirical incubation distributions and comparing disease incidence

We modelled variation in the Buruli ulcer incubation period by sampling incubation times from empirical minimum–maximum intervals for each case and simulating exposure dates across 200 Monte Carlo iterations. In each iteration, exposure dates were reconstructed by subtracting sampled incubation periods from observed onset dates, and cases were then grouped by epidemiological week and intervention arm (‘treatment’ or ‘control’). Weekly treatment−control differences and pre-/post-intervention summaries were computed for each iteration. A gamma distribution was fitted to the aggregated incubation samples, and 95% bootstrap confidence intervals (10,000 replicates) were obtained. Monte Carlo distributions of rate ratios and rate differences were used to derive uncertainty intervals for post-intervention effect estimates. We computed the mean incidence rate of infections that occurred during the trial period in the intervention or control group, taking the ‘relative risk’ as the ratio between the inferred incidence rates in intervention versus control arms. The null hypothesis was that there was no difference in Buruli ulcer infections between intervention and control arms during the evaluated trial period, as denoted by a RR = 1.

#### Identification of seasonal peaks in treatment and control zones

Weekly case densities derived from Monte Carlo exposure reconstructions for treatment and control zones were smoothed using a 50-week centred moving average to reduce short-term variability and highlight seasonal trends. Peak timing was identified separately for each zone and calendar year by locating the week with the maximum smoothed case density, representing the dominant seasonal peak. For each year, we calculated the temporal difference in seasonal peak timing between treatment and control zones as the number of days separating their respective peak dates.

#### Poisson likelihood ratio test

Event counts in treatment and control groups were modelled as independent Poisson processes with equal exposure. Differences in incidence rates were tested using a two-sided Poisson likelihood ratio test, with *P* values obtained from a *χ*^2^ distribution with one degree of freedom. The incidence rate ratio was estimated from maximum-likelihood rates, and 95% confidence intervals were calculated using a score-based method for the ratio of two Poisson rates. No adjustment for multiple comparisons was performed. Analyses were intended to assess intervention effects at distinct timepoints. Accordingly, reported *P* values are nominal and should be interpreted in conjunction with effect sizes and confidence intervals.

#### Visualizing case counts across day-wise sliding windows

We counted the number of cases with symptomatic onset 101–171 days after all possible exposure days from 1 January 2024 to 14 May 2024 (yielding a total of 136 IQR exposure windows: ‘sliding windows’). A person infected on unknown day *X* is expected to have a 50% chance of experiencing symptom onset between 101 and 171 days after day *X* (because the IQR accounts for 50% of the data within the incubation period probability distribution); a person infected on unknown day *X* + 1 similarly has a 50% chance of experiencing symptom onset between 101 and 171 days after *X* + 1 and so on. In this way, case counts in treatment and control zones were visually explored on a day-wise basis by running the same IQR temporal inclusion criteria across all days from 1 January 2024 to 14 May 2024, equating to 136 exposure days.

### Correlating mosquito population size changes with changes in Buruli ulcer case counts

For each exposure day, the control-minus-treatment case count difference (from each corresponding IQR exposure period time window) was calculated and aligned against the log-transformed mosquito egg counts (Supplementary Table 6). Here, the difference was calculated as the number of cases across all control zones minus the number of cases across all treatment zones for each exposure day. As egg collections were conducted at only 7 timepoints (3 before and 4 after the intervention), missing counts for all other IQR windows were imputed using cubic spline interpolation, implemented in the ‘scipy.interpolate’ Python library. To validate the imputation of egg count differences during the intervention period, the imputed data were compared, using a linear model, to observed data from a previous In2Care intervention (data collected during the intervention) conducted in a separate urban setting^24^. Following validation, the imputed egg counts were compared with the control-minus-treatment case count difference across all exposure days (IQR time windows) using time-series plots and linear modelling.

### Reporting summary

Further information on research design is available in the Nature Portfolio Reporting Summary linked to this article.

## Supplementary information


Supplementary InformationSupplementary Figs. 1–4.
Reporting Summary
Supplementary Tables 1–8Supplementary Tables 1–8.


## Data Availability

Source data are provided in Supplementary Tables 1–8. Specific accession numbers for sequences uploaded to NCBI GenBank can be found in Supplementary Table 8. All DNA sequencing data generated in this project are available under NCBI GenBank Bioproject ID PRJNA857537.

## Code availability

The code is available via GitHub at https://github.com/abuultjens/buruli-ulcer-mosquito-control-intervention (ref. ^40^) and Zenodo at https://doi.org/10.5281/zenodo.21799032 (ref. ^41^).
