# Supplementary material for: Autodissemination stations suppress Aedes notoscriptus mosquitoes and reduce Buruli ulcer risk in urban Australia: a randomized controlled field trial
Source: Nat Microbiol. 2026 Aug 27;11(9):2493–504. doi: 10.1038/s41564-026-02439-8 (PMC13524796; doi:10.1038/s41564-026-02439-8)
Supplement: Supplementary file 1 [file 41564_2026_2439_MOESM1_ESM.pdf]

nature microbiology

Supplementary information https://doi.org/10.1038/s41564-026-02439-8

Autodissemination stations suppress *Aedes notoscriptus* mosquitoes and reduce Buruli ulcer risk in urban Australia: a randomized controlled field trial

In the format provided by the authors and unedited

Supplementary Information

Autodissemination stations suppress *Aedes notoscriptus* mosquitoes and reduce risk of Buruli ulcer in urban Australia: a randomized controlled field trial

Véronique Paris, Andrew H. Buultjens, Nicholas Bell, Thomas L. Schmidt, Wendy M. Bamberg, Michael T. Birrell, Finn Romanes, Catarina A. Antao, Maria Globan, Caroline Lavender, Katherine Bond, Sally Dougall, Jake A. Lacey, Yichen Zhai, Peihong Guo, Norelle L. Sherry, Katherine B. Gibney, Jue Tao Lim, Paul D. R. Johnson, Timothy P. Stinear and Ary A. Hoffmann

Table of contents:

Figures:

Figure S1: Core genome phylogeny of 478 *M. ulcerans* clinical isolates

Figure S2: Information shared with residents of all intervention and control clusters

Figure S3: Recruitment leaflet for residents of all intervention clusters

Figure S4: Risk prevention information shared with residents of intervention and control clusters

Figure S1

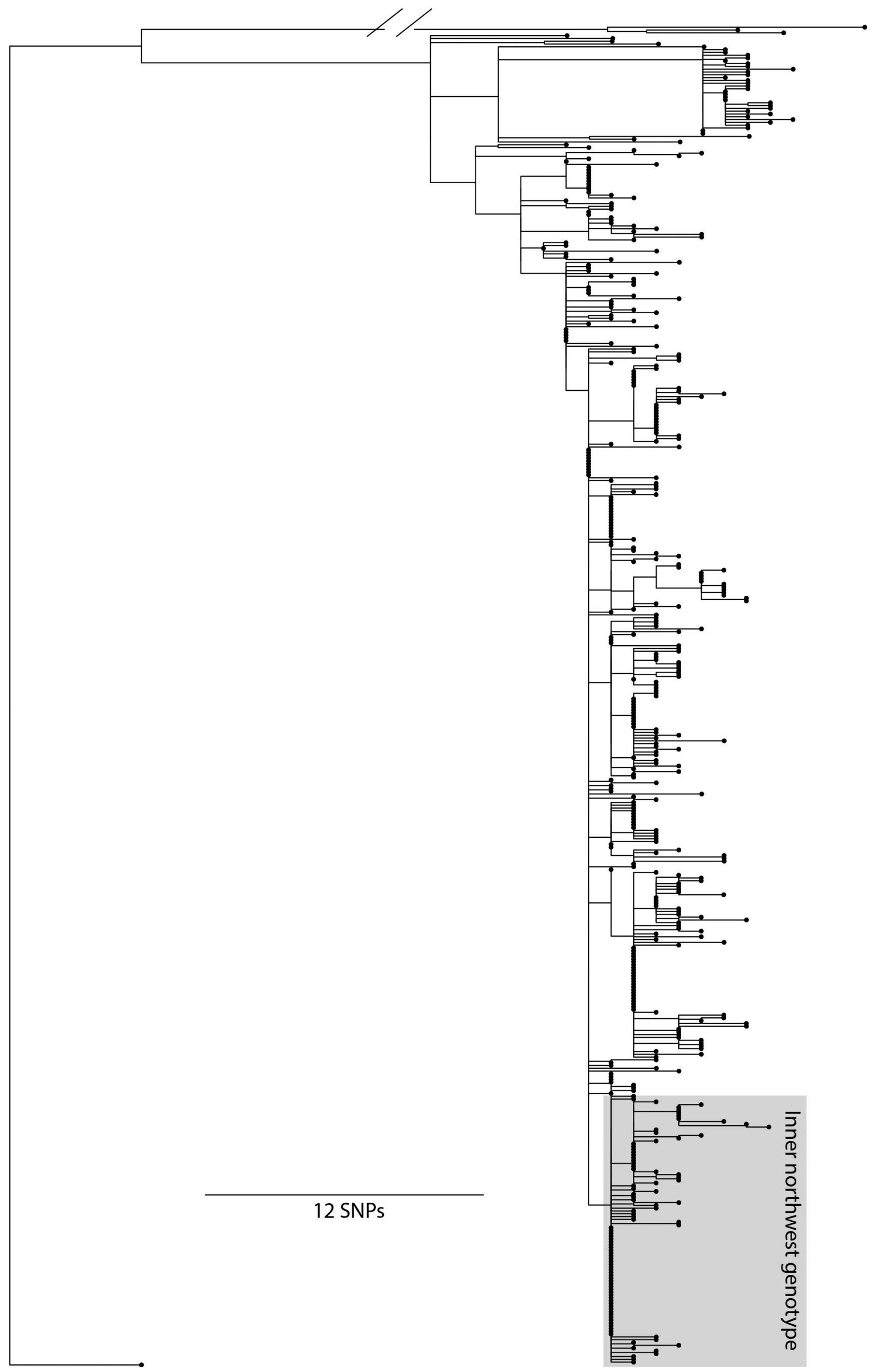

Figure S1: Core genome phylogeny of 478 *M. ulcerans* clinical isolates. Isolates were from patients in Victoria, showing clustering of inner-northwest isolate genomes (highlighted). Phylogeny inferred by maximum likelihood (FastTree v2.1.10, GTR model) using an alignment of core genome SNPs.

Figure S2

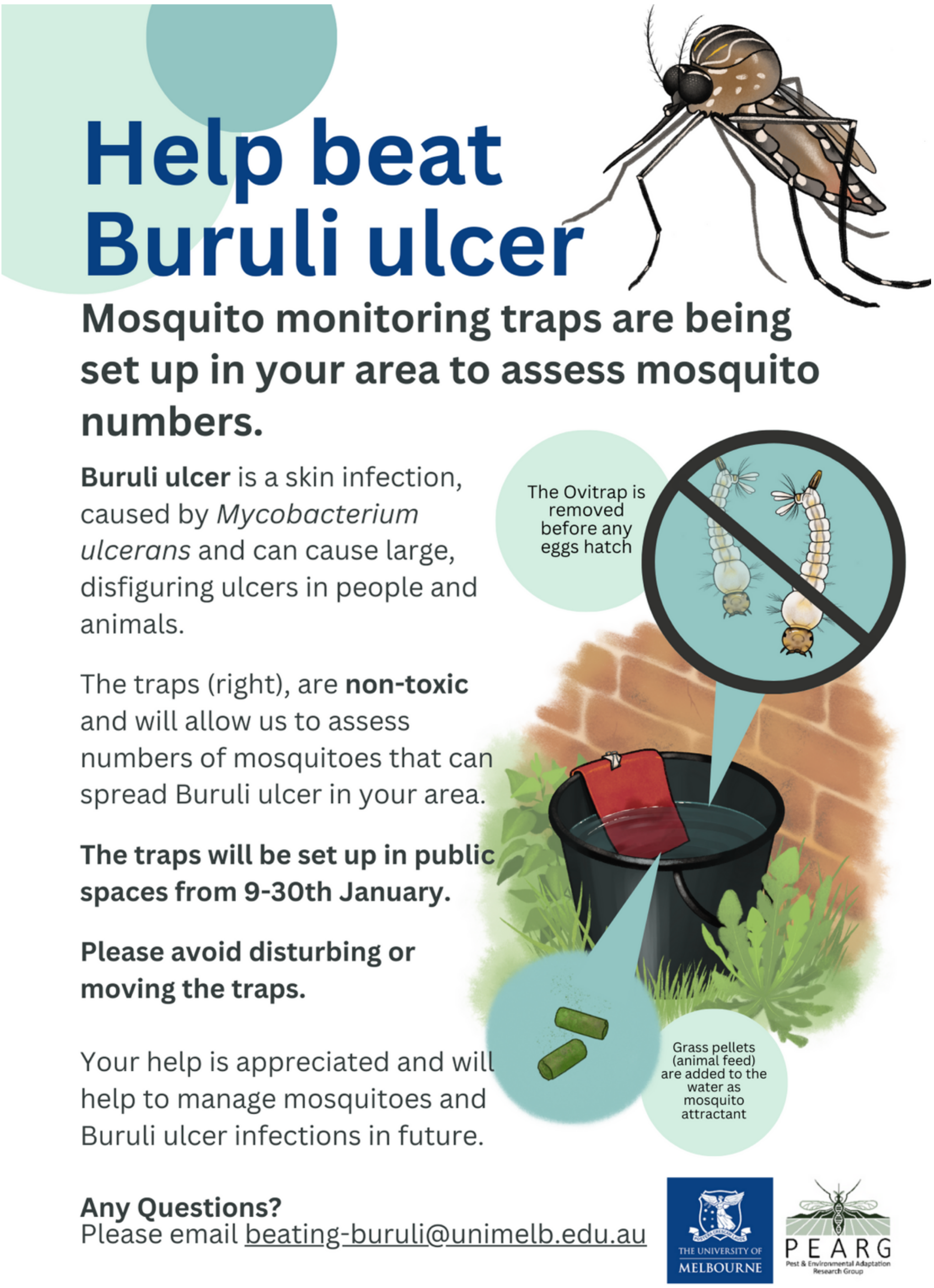

Figure S2: Information shared with residents of all intervention and control clusters - I. Leaflets were distributed to households to inform about the setup and function of ovitraps that were used for egg collections.

Figure S3

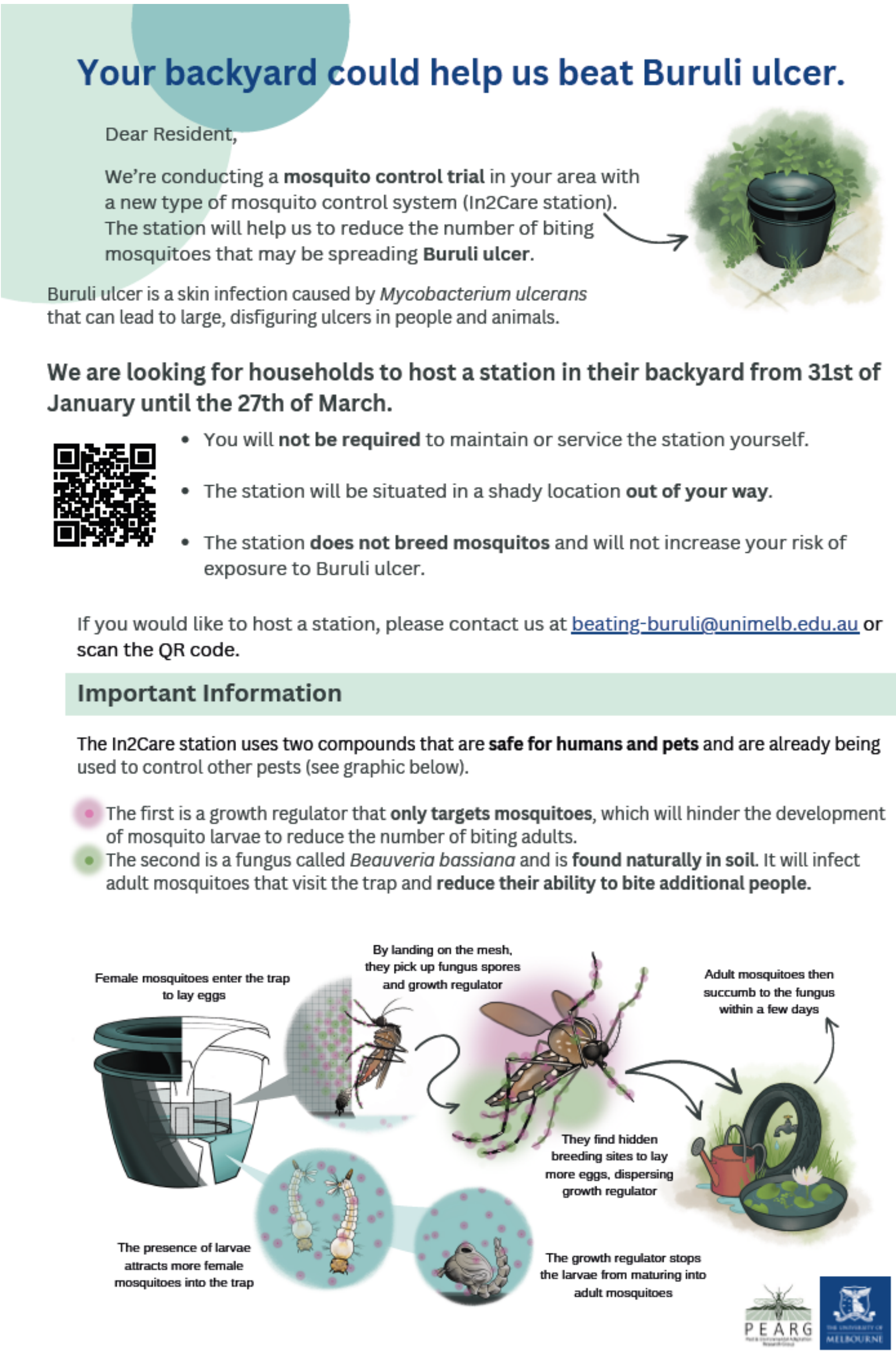

Your backyard could help us beat Buruli ulcer.

Dear Resident,

We're conducting a **mosquito control trial** in your area with a new type of mosquito control system (In2Care station). The station will help us to reduce the number of biting mosquitoes that may be spreading **Buruli ulcer**.

Buruli ulcer is a skin infection caused by *Mycobacterium ulcerans* that can lead to large, disfiguring ulcers in people and animals.

We are looking for households to host a station in their backyard from 31st of January until the 27th of March.

- You will **not be required** to maintain or service the station yourself.
- The station will be situated in a shady location **out of your way**.
- The station **does not breed mosquitos** and will not increase your risk of exposure to Buruli ulcer.

If you would like to host a station, please contact us at beating-buruli@unimelb.edu.au or scan the QR code.

Important Information

The In2Care station uses two compounds that are **safe for humans and pets** and are already being used to control other pests (see graphic below).

- The first is a growth regulator that **only targets mosquitoes**, which will hinder the development of mosquito larvae to reduce the number of biting adults.
- The second is a fungus called *Beauveria bassiana* and is **found naturally in soil**. It will infect adult mosquitoes that visit the trap and **reduce their ability to bite additional people.**

Figure S3: Recruitment leaflet for residents of all intervention clusters. Leaflets were distributed to households to inform about the functionality of the In2Care station and to recruit households to volunteer to host stations for the intervention period.

Figure S4

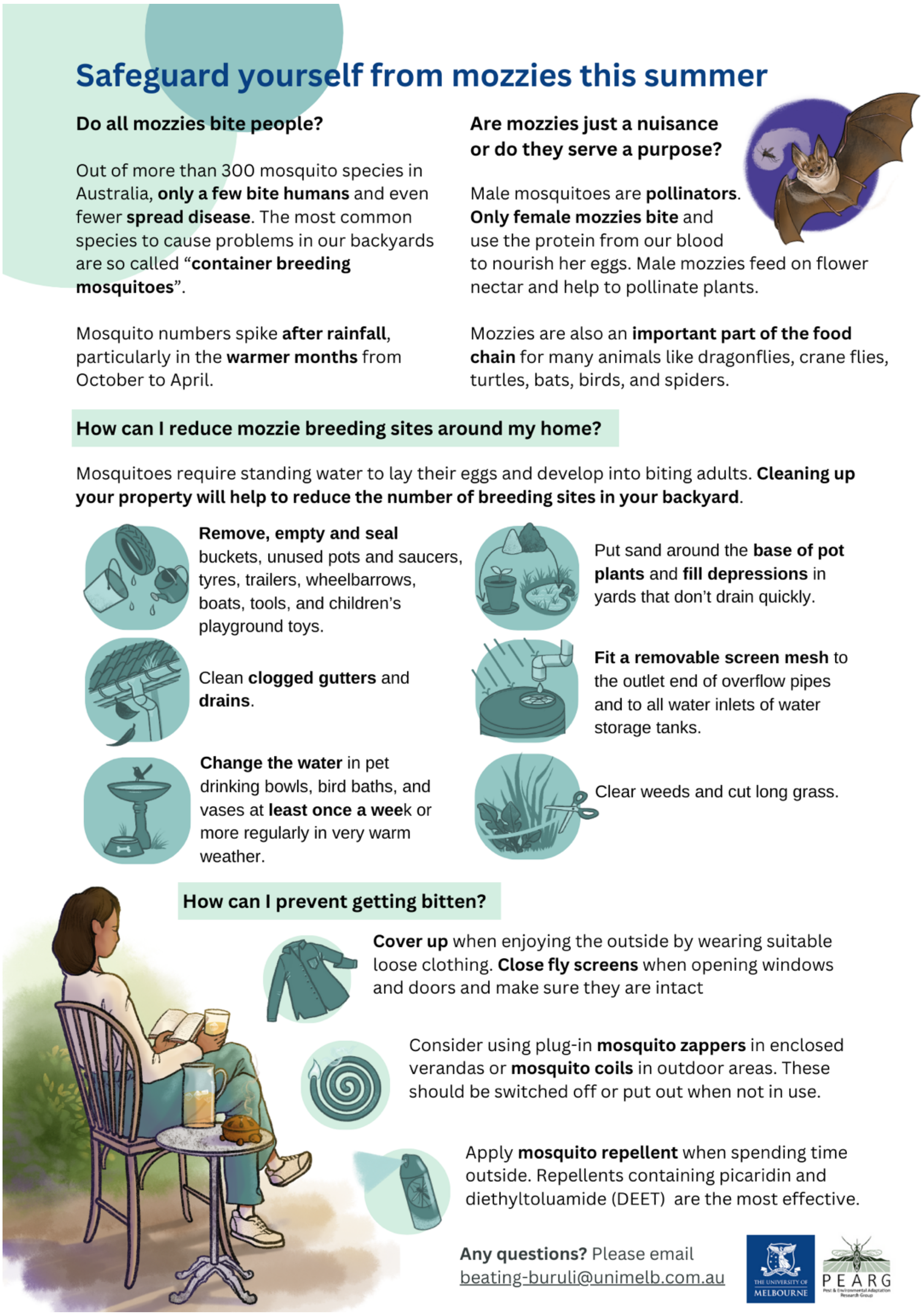

Figure S4: Risk prevention information shared with residents of intervention and control clusters. Leaflets were distributed to households to inform about mosquitoes and how to prevent mosquitoes from breeding in their property and how to prevent mosquito bites.
