# Supplementary material for: Autodissemination stations suppress Aedes notoscriptus mosquitoes and reduce Buruli ulcer risk in urban Australia: a randomized controlled field trial
Source: Nat Microbiol. 2026 Aug 27;11(9):2493–504. doi: 10.1038/s41564-026-02439-8 (PMC13524796; doi:10.1038/s41564-026-02439-8)
Supplement: Supplementary file 2 [file 41564_2026_2439_MOESM2_ESM.pdf]

nature portfolio

Corresponding author(s): Véronique Paris, Timothy Stinear

Last updated by author(s): Aug 12, 2025

Reporting Summary

Nature Portfolio wishes to improve the reproducibility of the work that we publish. This form provides structure for consistency and transparency in reporting. For further information on Nature Portfolio policies, see our Editorial Policies and the Editorial Policy Checklist.

Statistics

For all statistical analyses, confirm that the following items are present in the figure legend, table legend, main text, or Methods section.

n/a	Confirmed	
☐	☒	The exact sample size (*n*) for each experimental group/condition, given as a discrete number and unit of measurement
☐	☒	A statement on whether measurements were taken from distinct samples or whether the same sample was measured repeatedly
☐	☒	The statistical test(s) used AND whether they are one- or two-sided<br>*Only common tests should be described solely by name; describe more complex techniques in the Methods section.*
☐	☒	A description of all covariates tested
☐	☒	A description of any assumptions or corrections, such as tests of normality and adjustment for multiple comparisons
☐	☒	A full description of the statistical parameters including central tendency (e.g. means) or other basic estimates (e.g. regression coefficient) AND variation (e.g. standard deviation) or associated estimates of uncertainty (e.g. confidence intervals)
☐	☒	For null hypothesis testing, the test statistic (e.g. F, t, r) with confidence intervals, effect sizes, degrees of freedom and P value noted<br>*Give P values as exact values whenever suitable.*
☒	☐	For Bayesian analysis, information on the choice of priors and Markov chain Monte Carlo settings
☐	☒	For hierarchical and complex designs, identification of the appropriate level for tests and full reporting of outcomes
☒	☐	Estimates of effect sizes (e.g. Cohen's *d*, Pearson's *r*), indicating how they were calculated

Our web collection on statistics for biologists contains articles on many of the points above.

Software and code

Policy information about availability of computer code

Data collection	All data used in the manuscript are available in the supplementary and source materials.
Data analysis	All analysis code is posted here: https://github.com/abuultjens/buruli-ulcer-mosquito-control-intervention

For manuscripts utilizing custom algorithms or software that are central to the research but not yet described in published literature, software must be made available to editors and reviewers. We strongly encourage code deposition in a community repository (e.g. GitHub). See the Nature Portfolio guidelines for submitting code & software for further information.

Data

Policy information about availability of data

All manuscripts must include a data availability statement. This statement should provide the following information, where applicable:

- Accession codes, unique identifiers, or web links for publicly available datasets
- A description of any restrictions on data availability
- For clinical datasets or third party data, please ensure that the statement adheres to our policy

Data availability: Source data are provided with this publication. DNA sequencing data generated in this project are available under the following NCBI GenBank Bioproject ID: PRJNA857537.
Code availability:The code is available via GitHub at https://github.com/abuultjens/buruli-ulcer-mosquito-control-intervention.
Snippy v4.4.5 (minfrac 0.9 and mincov 10) (https://github.com/tseemann/snippy)

FastTree v2.1.10 (https://doi.org/10.1093/molbev/msp077)
Lenth R, Piaskowski J (2026). emmeans: Estimated Marginal Means, aka Least-Squares Means. R package version 2.0.3, https://rvlenth.github.io/emmeans/.
glmmTMB v1.1.14 Mollie E. Brooks, Kasper Kristensen, Koen J. van Benthem, Arni Magnusson, Casper W. Berg, Anders Nielsen, Hans J. Skaug, Martin Maechler and Benjamin M. Bolker (2017). glmmTMB Balances Speed and Flexibility Among Packages for Zero-inflated Generalized Linear Mixed Modeling. The R Journal, 9(2), 378-400. doi: 10.32614/RJ-2017-066. https://github.com/glmmTMB/glmmTMB

Research involving human participants, their data, or biological material

Policy information about studies with human participants or human data. See also policy information about sex, gender (identity/presentation), and sexual orientation and race, ethnicity and racism.

Reporting on sex and gender	This study did not involve human participants. The use of de-identified human Buruli ulcer case location data was approved by the ethics committee listed below.
Reporting on race, ethnicity, or other socially relevant groupings	This study did not have human participants. The use of de-identified human Buruli ulcer case location data was approved by the ethics committees listed below.
Population characteristics	This study did not have human participants . The use of de-identified human Buruli ulcer case location was approved by the ethics committees listed below.
Recruitment	This study did not have human participants . The use of de-identified human Buruli ulcer case location was approved by the ethics committees listed below.
Ethics oversight	Ethical approval for the use in this study of de-identified human Buruli ulcer case location data, aggregated at the mesh block level, was obtained from Victorian Government Department of Health Human Ethics Committee under HREC/54166/DHHS-2019-179235 and from the University of Melbourne Human Research Ethics Committee under 2024-31315-60974-3. Mesh blocks are the smallest geographic areas defined by the Australian Bureau of Statistics, and usually contain 30 to 60 dwellings

Note that full information on the approval of the study protocol must also be provided in the manuscript.

Field-specific reporting

Please select the one below that is the best fit for your research. If you are not sure, read the appropriate sections before making your selection.

☐ Life sciences ☐ Behavioural & social sciences ☒ Ecological, evolutionary & environmental sciences

For a reference copy of the document with all sections, see nature.com/documents/nr-reporting-summary-flat.pdf

Ecological, evolutionary & environmental sciences study design

All studies must disclose on these points even when the disclosure is negative.

Study description	We designed a randomized controlled trial in an urban Buruli ulcer endemic area in Melbourne, Australia to test if autodissemination mosquito control stations supress Ae. notoscriptus populations (primary outcome). We also examined the impact of mosquito suppression on notified cases of Buruli ulcer (secondary outcome). We randomly assigned six geographic clusters to each receive 100 autodissemination stations for eight weeks (intervention clusters) and six clusters to receive no stations (control clusters). The stations contained pyriproxyfen and the entomopathogenic fungus Beauveria bassiana.
Research sample	The outcome of the mosquito intervention was assessed by collecting and counting mosquito eggs collected through oviposition traps. The outcome of the mosquito intervention on Buruli ulcer transmission to humans was assessed by comparing Buruli ulcer case notification data in treatment clusters with control clusters.
Sampling strategy	Population density of Ae. notoscriptus was evaluated using egg counts collected through ovitraps at control and treatment clusters from the 9th to the 23rd of January 2024 for baseline before intervention egg counts and then again from the 28th of March until the 18th of April 2024 for the after-intervention egg counts. A total of 480 ovitraps (40 per site) were used, each consisting of a 500 mL bucket half-filled with water and alfalfa pellets to attract gravid mosquitoes (Figure 1B). A 15 cm red felt strip provided an oviposition site. Eggs were counted weekly using a light microscope by the same researcher for consistency. For assessing the impact of the intervention on Buruli ulcer transmission to humans, the notified cases of Buruli ulcer were obtained from the Victorian State Government Department of Health, de-identified and aggregated at mesh block level, after adjusting for the mean 4.8-month incubation period.
Data collection	After the eggs were collected from the field, they were counted using a light microscope and entered into the egg count data sheet. For Buruli ulcer human case notification data, information was obtained from the Victorian State Government Department of Health for the period of the study.
Timing and spatial scale	Egg counts were collected for three weeks from the 9th to the 23rd of January 2024 and then again from the 28th of March until the 18th of April. For Buruli ulcer human case notification data, we first estimated likely exposure dates by accounting for the long incubation period. We considered Buruli ulcer cases that occurred within the reported inter-quartile range (IQR) of the incubation

	period 101-171 days after all possible exposure days - from the 1st of January 2024 to the 14th of May 2024. This yielded a total of 136 exposure days, corresponding with 136 IQR symptom onset windows, meaning cases were counted within a notification range from the 11th April 2024 - 24th August 2024.
Data exclusions	For the mosquito egg counts, no data was excluded. For Buruli ulcer human case notification data, only cases confirmed by pathogen genomic epidemiology as originating in the study area were included. All other case notifications were excluded.
Reproducibility	Reproducibility was assured (in part) with 12 clusters across the study area, six assigned as control and six assigned as intervention clusters.
Randomization	In this interventional study, we randomly assigned geographic areas using computer-generated random numbers to six treatment clusters and six control clusters.
Blinding	There was no blinding in the mosquito egg count analysis. There was no blinding for the human case notification analysis, but cases were counted in control and treatment areas against defined, objective criteria, equally applied across the study area, with no subjective assessment or interpretation.

Did the study involve field work? ☒ Yes ☐ No

Field work, collection and transport

Field conditions	The field intervention took place in Melbourne summer 23/24. Melbourne experiences wet an humid conditions from December to February with an average rainfall of ~5mm per month and an average max temperature of 26 degrees Celsius. Rainfall then dropped to almost 0mm for the months of February and March, while the average max temperature increased to 28 degrees Celsius. Temperatures then decreased to below 20 degrees Celsius for the months of April and May.
Location	Inner-northwest suburbs of Melbourne including postcodes: 3032, 3039, 3040, 3041, 3042, 3044, 3055, 3058
Access & import/export	All egg collections were performed on public land (mostly nature strips). Intervention mosquito stations were placed on public land as well as in residential properties. We acquired permission of the property owner prior to installing traps at their property.
Disturbance	Materials were deployed in the described areas and we minimized disturbance of those materials by carrying out frequent checks to ensure material moved by weather, people or animals were either removed or reinstated.

Reporting for specific materials, systems and methods

We require information from authors about some types of materials, experimental systems and methods used in many studies. Here, indicate whether each material, system or method listed is relevant to your study. If you are not sure if a list item applies to your research, read the appropriate section before selecting a response.

Materials & experimental systems

n/a	Involved in the study	
☒	☐	Antibodies
☒	☐	Eukaryotic cell lines
☒	☐	Palaeontology and archaeology
☐	☒	Animals and other organisms
☒	☐	Clinical data
☒	☐	Dual use research of concern
☒	☐	Plants

Methods

n/a	Involved in the study	
☒	☐	ChIP-seq
☒	☐	Flow cytometry
☒	☐	MRI-based neuroimaging

Animals and other research organisms

Policy information about studies involving animals; ARRIVE guidelines recommended for reporting animal research, and Sex and Gender in Research

Laboratory animals	The study did not involve laboratory animals
Wild animals	The study collected mosquito eggs as a proxy for mosquito abundance.
Reporting on sex	Eggs were not reared to adults, we therefore have no reporting on the sec of collected mosquitoes.
Field-collected samples	Mosquito eggs were collected and frozen after that they were counted.
Ethics oversight	No ethics approval is needed for this work.

Note that full information on the approval of the study protocol must also be provided in the manuscript.

Plants

Seed stocks	No plants were used in this study.
Novel plant genotypes	No plants were used in this study.
Authentication	No plants were used in this study.
